# Hydrogels of Polycationic Acetohydrazone-Modified Phosphorus Dendrimers for Biomedical Applications: Gelation Studies and Nucleic Acid Loading

**DOI:** 10.3390/pharmaceutics10030120

**Published:** 2018-08-06

**Authors:** Evgeny K. Apartsin, Alina E. Grigoryeva, Audrey Malrin-Fournol, Elena I. Ryabchikova, Alya G. Venyaminova, Serge Mignani, Anne-Marie Caminade, Jean-Pierre Majoral

**Affiliations:** 1 Institute of Chemical Biology and Fundamental Medicine SB RAS, 8, Lavrentiev ave., 630090 Novosibirsk, Russia; feabelit@mail.ru (A.E.G.); lenryab@niboch.nsc.ru (E.I.R.); ven@niboch.nsc.ru (A.G.V.); 2 Ecole des Mines d’Albi Carmaux, Campus Jarlard, 81000 Albi, France; amalrinf@mines-albi.fr; 3 Laboratoire de Chimie et de Biochimie Pharmacologiques et Toxicologiques, Université Paris Descartes, PRES Sorbonne Paris Cité, CNRS UMR 8601, 75006 Paris, France; serge.mignani@parisdescartes.fr; 4 Centro de Química da Madeira, MMRG, Universidade da Madeira, 9000-390 Funchal, Portugal; 5 Laboratoire de Chimie de Coordination du CNRS, 205, route de Narbonne, BP 44099, 31077 Toulouse CEDEX 04, France; anne-marie.caminade@lcc-toulouse.fr; 6 LCC-CNRS, Université de Toulouse, CNRS, 31013 Toulouse, France

**Keywords:** phosphorus dendrimers, functionalization, Girard reagents, hydrogels, oligonucleotides

## Abstract

In this work, we report the assemblage of hydrogels from phosphorus dendrimers in the presence of biocompatible additives and the study of their interactions with nucleic acids. As precursors for hydrogels, phosphorus dendrimers of generations 1–3 based on the cyclotriphosphazene core and bearing ammonium or pyridinium acetohydrazones (Girard reagents) on the periphery have been synthesized. The gelation was done by the incubation of dendrimer solutions in water or phosphate-buffered saline in the presence of biocompatible additives (glucose, glycine or polyethylene glycol) to form physical gels. Physical properties of gels have been shown to depend on the gelation conditions. Transmission electron microscopy revealed structural units and well-developed network structures of the hydrogels. The hydrogels were shown to bind nucleic acids efficiently. In summary, hydrogels of phosphorus dendrimers represent a useful tool for biomedical applications.

## 1. Introduction

Local drug delivery is a powerful approach to treat a wide number of diseases, including cancer, inflammation and so forth. Indeed, the targeted application of therapeutic permits to achieve locally high concentration of a drug, whereas avoiding potential systemic toxicity, drug-drug interactions, side effects and related complications [1]. Hydrogels are considered prospective drug carriers for local delivery. Indeed, hydrogels can be prepared by simple procedures, they possess high drug loading capacity; by optimizing the chemical structure of precursor molecules, hydrogels can be made non-toxic and biodegradable. Being abundantly hydrated, hydrogels can be easily applied to tissues and organs of choice [2,3,4].

Dendrimers—symmetric hyperbranched polymers—are considered prospective tools for nanomedicine [5]. In particular, they have been recently used as building blocks for hydrogels. Choosing the architecture of dendrimers, one can program the properties and structure of hydrogels, such as reversibility (physical or chemical gels [6]), self-assembly patterns [7] and so forth. Recently, one dendrimer-containing gel drug, Vivagel^®^, has been approved by the US FDA for the use in condom lubricants to prevent the HIV infection [8,9]. It is subjected for clinical trials as a therapeutic to treat bacterial vaginosis as well [10,11]. This recent success encourages the development of new dendrimer-containing constructions and materials for innovative medicine.

Among the wide variety of dendrimer architectures, phosphorus dendrimers represent a unique object for nanomedicine [12]. They possess biological activity per se (such as anti-cancer [13,14], immunostimulatory [15] and anti-prion [16] as well as act as efficient carriers for plasmids [17] or therapeutic nucleic acids [18,19,20,21]. The ability of phosphorus dendrimers to be both structural block and functional element of a therapeutic construction can be fully realized in the design of dendrimer-based hydrogels. Such hydrogels can be a promising material for tissue engineering and drug delivery. However, till now, known examples of phosphorus dendrimers-based hydrogels [22] were mainly prepared using additives that could be cytotoxic when taken in high concentrations. Obviously, this approach needs optimization to produce novel biomaterials for medicine.

Herein, we report the assemblage of hydrogels of phosphorus dendrimers formed in the presence of biocompatible precursors, study of their structural features and loading with a macromolecular cargo (model oligonucleotide).

## 2. Materials and Methods

### 2.1. Chemicals

Glucose and glycine (biotechnology grade) were supplied by Helicon, Moscow, Russia. Polyethylene glycol (average molecular weight 4000, for synthesis), trimethylammonium chloride acetohydrazide (Girard reagent T, 98%), pyridinium chloride acetohydrazide (Girard reagent P, 99%) were from Sigma-Aldrich, Hamburg, Germany. Phosphate-buffered saline, PBS (10 mM sodium phosphate, 2.7 mM KCl, 137 mM NaCl, pH 7.4), was from Gibco, Thermo Fischer Scientific, Waltham, MA USA. Water solutions were prepared using milliQ deionized water (18.2 MΩ/cm) obtained by Millipore Simplicity water purification system (Millipore, Burlington, MA, USA).

Oligonucleotides were synthesized using the solid-phase phosphoramidite method in an ASM-800 automated synthesizer (Biosset, Novosibirsk, Russia) from commercially available phosphoramidites (Glen Research, Sterling, VA, USA) according to the protocols optimized for the given equipment and purified by 15% PAGE assay.

### 2.2. Synthesis of Girard T, P-Modified Phosphorus Dendrimers

All manipulations were carried out using standard dry argon-high vacuum technique. Organic solvents were dried and distilled prior to use. Aldehyde-terminated phosphorus dendrimers of generations 1–3 were obtained by growing from a cyclotriphosphazene core as described in [23]. Girard T or P-modified dendrimers were synthesized according to the procedure reported for the modification of dendrimers having thiophosphate as a core [22].

**Figure 1:**
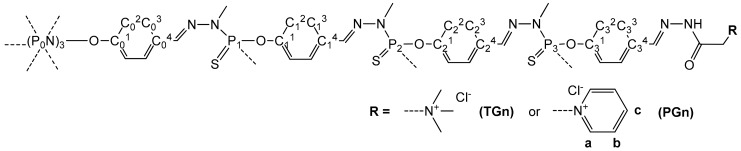
Atom numbering used for the NMR signal attribution.

^1^H, ^13^C{^1^H} and ^31^P{^1^H} NMR spectra were recorded using AV400PAS, AV400LIQ spectrometers (Bruker, Karlsruhe, Germany). The attribution of the NMR signals of the dendrimer branches was made by analogy with Refs. [23,24], the attribution of the NMR signals of the amines on the periphery was made by analogy with Ref. [22]. To assign the ^13^C NMR signals, Jmod, HMBC, HBQC NMR experiments were additionally done, if necessary. The atom numbering used for the signals attribution is given in Figure 1. Mass spectrometry was not used to prove the purity of these dendrimers because of spontaneous rearrangements in the phosphorhydrazone structure during the analysis [25], thus, the purity of the dendrimer samples was assessed only by NMR [26].

#### Grafting of Girard Reagents onto the Periphery of Dendrimers: General Procedure

To a solution of aldehyde-terminated dendrimer Gn (300 mg, 0.1 mmol (G1), 0.044 mmol (G2), 0.02 mmol (G3)) in 20 mL chloroform, a solution of Girard reagent T or P (1.05 eq. per aldehyde function, 1.26 mmol (G1), 1.11 mmol (G2), 1.01 mmol (G3)) in 10 mL methanol was added dropwise upon stirring. Several drops of acetic acid were added to reach the pH ~5. Reaction mixture was stirred overnight at room temperature. The completeness of conversion was proven by the disappearance of free aldehyde signals in ^1^H NMR (DMSO-d6) at 9.98 ppm. The reaction mixture was evaporated to dryness, the solid residue was washed with chloroform (25 mL), diethyl ether (2 × 25 mL) and dried. Girard T or P-modified dendrimers were obtained as pale-yellow powders in quantitative yield.

*Dendrimer **TG1***. ^1^H NMR (400 MHz, DMSO-d6) δ 4.46, 4.85 (s, 24 H, CH_2_), 7.02 (d, *J* = 7.6, 12 H, C_0_^3^H), 7.20 (d, *J* = 8.0, 24 H, C_1_^3^H), 7.68 (m, 36 H, C_0_^2^H, C_1_^2^H), 8.05 (s, 6 H, C_0_^4^-CH), 8.16, 8.36 (s, 12 H, C_1_^4^-CH), 12.32, 13.19 (br. s, 12 H, N-NH-C(O)). ^13^C{^1^H} NMR (101 MHz, DMSO-d6) δ 33.5 (d, *J* = 13.5, CH_3_NP_1_), 53.8 (d, *J* = 13.5, N^+^(CH_3_)_3_), 62.7, 63.8 (s, CH_2_), 121.9 (m, C_0_^3^, C_1_^3^), 129.3 (m, C_0_^2^, C_1_^2^), 131.8 (s, C_1_^4^), 132.6 (s, C_0_^4^), 141.4 (s, CH=NNP_1_), 144.6, 148.0 (s, CH=NNH), 151.1 (m, C_0_^1^, C_1_^1^), 160.4, 165.8 (s, N-NH-C(O)). ^31^P{^1^H} NMR (162 MHz, DMSO-d6) δ 8.4 (s, P_0_), 62.1 (s, P_1_). Isomer ratio: 65:35.

*Dendrimer **TG2***. ^1^H NMR (400 MHz, DMSO-d6) δ 4.47, 4.85 (s, 48 H, CH_2_), 6.97 (d, *J* = 7.6, 12 H, C_0_^3^H), 7.21 (m, 72 H, C_1_^3^H, C_2_^3^H), 7.69 (m, 84 H, C_0_^2^H, C_1_^2^H, C_2_^2^H), 7.98 (m, 18 H, C_0_^4^-CH, C_1_^4^-CH), 8.20, 8.38 (s, 24 H, C_2_^4^-CH), 12.37, 13.23 (br. s, 24 H, N-NH-C(O)). ^13^C{^1^H} NMR (101 MHz, DMSO-d6) δ 33.5 (d, *J* = 13.5, CH_3_NP_1_, CH_3_NP_1_), 53.8 (d, *J* = 13.5, N^+^(CH_3_)_3_), 62.7, 63.8 (s, CH_2_), 121.9 (m, C_0_^3^, C_1_^3^, C_2_^3^), 129.2 (m, C_0_^2^, C_1_^2^, C_2_^2^), 131.5 (s, C_1_^4^, C_2_^4^), 132.6 (s, C_0_^4^), 141.5 (s, CH=NNP_1_, CH=NNP_2_), 144.6, 148.1 (s, CH=NNH), 151.7 (m, C_0_^1^, C_1_^1^, C_2_^1^), 160.5, 166.0 (s, N-NH-C(O)). ^31^P{^1^H} NMR (162 MHz, DMSO-d6) δ 8.5 (s, P_0_), 62.3 (m, P_1_, P_2_). Isomer ratio: 63:37.

*Dendrimer **TG3***. ^1^H NMR (400 MHz, DMSO-d6) δ 4.48, 4.84 (s, 96 H, CH_2_), 7.21 (m, 180 H, C_0_^3^H, C_1_^3^H, C_2_^3^H, C_3_^3^H), 7.71 (m, 180 H, C_0_^2^H, C_1_^2^H, C_2_^2^H, C_3_^2^H), 7.99 (m, 42 H, C_0_^4^-CH, C_1_^4^-CH, C_2_^4^-CH), 8.20, 8.38 (s, 48 H, C_2_^4^-CH), 12.36, 13.22 (br. s, 48 H, N-NH-C(O)). ^13^C{^1^H} NMR (101 MHz, DMSO-d6) δ 33.5 (m, CH_3_NP_1_, CH_3_NP_1_, CH_3_NP_2_), 53.9 (d, *J* = 13.5, N^+^(CH_3_)_3_), 62.7, 63.8 (s, CH_2_), 121.9 (m, C_0_^3^, C_1_^3^, C_2_^3^, C_3_^3^), 129.1 (m, C_0_^2^, C_1_^2^, C_2_^2^, C_3_^2^), 131.5 (s, C_0_^4^, C_1_^4^, C_2_^4^, C_3_^4^), 132.5 (s, C_0_^4^), 141.3 (s, CH=NNP_1_, CH=NNP_2_, CH=NNP_3_), 144.8, 148.1 (s, CH=NNH), 151.7 (m, C_0_^1^, C_1_^1^, C_2_^1^, C_3_^1^), 160.3, 166.1 (s, N-NH-C(O)). ^31^P{^1^H} NMR (162 MHz, DMSO-d6) δ 8.1 (s, P_0_), 62.0 (m, P_1_, P_2_, P_3_). Isomer ratio: 65:35.

*Dendrimer **PG1***. ^1^H NMR (400 MHz, DMSO-d6) δ 5.61, 5.76 (s, 24 H, CH_2_), 6.99 (d, *J* = 7.6, 12 H, C_0_^3^H), 7.23 (d, *J* = 8.0, 24 H, C_1_^3^H), 7.71 (m, 36 H, C_0_^2^H, C_1_^2^H), 8.10 (s, 6 H, C_0_^4^-CH), 8.19 (s, 24 H, C_a_H), 8.22, 8.40 (s, 12 H, C_1_^4^-CH), 8.68 (t, *J* = 7.8, 12 H, C_c_H), 9.14 (s, 24 H, C_b_H), 12.48, 13.26 (br. s, 12 H, N-NH-C(O)). ^13^C{^1^H} NMR (101 MHz, DMSO-d6) δ 33.6 (d, *J* = 13.5, CH_3_NP_1_), 61.4, 61.8 (s, CH_2_), 121.9 (m, C_0_^3^, C_1_^3^), 128.0 (C_a_), 129.0 (m, C_0_^2^, C_1_^2^), 131.7 (s, C_1_^4^), 132.6 (s, C_0_^4^), 141.4 (s, CH=NNP_1_), 144.5, 146.9 (s, CH=NNH), 147.3 (m, C_b_, C_c_), 151.7 (m, C_0_^1^, C_1_^1^), 161.9, 166.9 (s, N-NH-C(O)). ^31^P{^1^H} NMR (162 MHz, DMSO-d6) δ 8.4 (s, P_0_), 62.1 (s, P_1_). Isomer ratio: 77:23.

*Dendrimer **PG2***. Synthesis of this dendrimer has been described previously in [22]. ^1^H NMR (400 MHz, DMSO-d6) δ 5.77, 6.10 (s, 48 H, CH_2_), 6.97 (s, 12 H, C_0_^3^H), 7.23 (m, 72 H, C_1_^3^H, C_2_^3^H), 7.66 (m, 12 H, C_0_^2^H), 7.72 (m, 72 H, C_1_^2^H, C_2_^2^H), 8.00 (m, 18 H, C_0_^4^-CH, C_1_^4^-CH), 8.19 (s, 48 H, C_a_H), 8.26, 8.41 (s, 24 H, C_2_^4^-CH), 8.65 (t, *J* = 7.8, 12 H, C_c_H), 9.15 (s, 24 H, C_b_H), 12.52, 13.33 (br. s, 24 H, N-NH-C(O)). ^13^C{^1^H} NMR (101 MHz, DMSO-d6) δ 33.5 (d, *J* = 13.5, CH_3_NP_1_, CH_3_NP_1_), 61.8, 65.5 (s, CH_2_), 121.9 (m, C_0_^3^, C_1_^3^, C_2_^3^), 128.0 (C_a_), 129.0 (m, C_0_^2^, C_1_^2^, C_2_^2^), 131.9 (s, C_1_^4^, C_2_^4^), 132.6 (s, C_0_^4^), 141.7 (s, CH=NNP_1_, CH=NNP_2_), 144.5, 146.9 (s, CH=NNH), 147.3 (m, C_b_, C_c_), 151.8 (m, C_0_^1^, C_1_^1^, C_2_^1^), 161.9, 166.9 (s, N-NH-C(O)). ^31^P{^1^H} NMR (162 MHz, DMSO-d6) δ 8.3 (s, P_0_), 62.3 (m, P_1_, P_2_). Isomer ratio: 76:24.

*Dendrimer **PG3***. ^1^H NMR (400 MHz, DMSO-d6) δ 5.77, 6.09 (s, 96 H, CH_2_), 7.23 (m, 180 H, C_0_^3^H, C_1_^3^H, C_2_^3^H, C_3_^3^H), 7.72 (m, 180 H, C_0_^2^H, C_1_^2^H, C_2_^2^H, C_3_^2^H), 8.00 (m, 42 H, C_0_^4^-CH, C_1_^4^-CH, C_2_^4^-CH), 8.17 (s, 96 H, C_a_H), 8.26, 8.41 (s, 48 H, C_2_^4^-CH), 8.63 (m, 48 H, C_c_H), 9.15 (s, 96 H, C_b_H), 12.54, 13.28 (br. s, 48 H, N-NH-C(O)). ^13^C{^1^H} NMR (101 MHz, DMSO-d6) δ 33.6 (m, CH_3_NP_1_, CH_3_NP_1_, CH_3_NP_2_), 60.6, 61.8 (s, CH_2_), 121.9 (m, C_0_^3^, C_1_^3^, C_2_^3^, C_3_^3^), 127.9 (C_a_), 129.1 (m, C_0_^2^, C_1_^2^, C_2_^2^, C_3_^2^), 131.7 (s, C_0_^4^, C_1_^4^, C_2_^4^, C_3_^4^), 132.7 (s, C_0_^4^), 141.6 (s, CH=NNP_1_, CH=NNP_2_, CH=NNP_3_), 144.5, 146.9 (s, CH=NNH), 147.3 (m, C_b_, C_c_), 151.8 (m, C_0_^1^, C_1_^1^, C_2_^1^, C_3_^1^), 161.8, 166.9 (s, N-NH-C(O)). ^31^P{^1^H} NMR (162 MHz, DMSO-d6) δ 8.1 (s, P_0_), 62.2 (m, P_1_, P_2_, P_3_). Isomer ratio: 79:21.

### 2.3. Preparation of Hydrogels

10 mg of a dendrimer **TGn** or **PGn** was dissolved in 500 μL of a dispersant upon vortexing and incubated in a 1.5 mL Eppendorf type plastic test tube at 65 °C. As a dispersant, the following were used: deionized water; PBS; 10% glucose (Glc) in deionized water or PBS; 10% glycine (Gly) in deionized water or PBS; 10% polyethylene glycol-4000 (PEG) in deionized water or PBS. The gelation was considered finished when the appearance of gels in test tubes ceased to change. Depending on the physical properties of a final gel, the gelation was considered complete (rigid, coherent gel), incomplete (gel and turbid supernatant) or partial (incoherent gel).

### 2.4. Transmission Electron Microscopy

Hydrogels were visualized in transmission electron microscopy (TEM) using two approaches: negative staining of native gels and ultrathin sections. Copper TEM grids covered with formvar film preliminary stabilized with carbon evaporation were used for the sample adsorption.

To prepare negatively stained samples, semifluid hydrogels were adsorbed on a TEM grid for 1 min. In the case of more rigid hydrogels, a piece of about 2 mm^3^ was mixed with 2 μL of distilled water using a needle. Then, a grid was placed onto gel for 1 min, after that visible pieces of gel were gently removed by a needle. The grids with adsorbed samples of hydrogels were contrasted for 10 s on a drop of 2% phosphotungstic acid (pH 0.5). At least 5–6 grid-fields were examined for each sample in TEM.

To prepare ultrathin sections, hydrogels fixed in 4% paraformaldehyde were postfixed in 1% OsO_4_, routinely dehydrated in ethanol and acetone and embedded in epon-araldit mixture. Ultrathin sections were prepared on a Leica EM UC7 ultratome (Leica Microsystems, Wein, Austria) and routinely contrasted with uranyl acetate and led citrate. All grids were examined in JEM 1400 TEM (JEOL, Tokyo, Japan), digital images were collected with a Veleta camera (EMSIS, Muenster, Germany). At least 10 individual ultrathin sections of each sample were examined in TEM.

### 2.5. Fluorescence Intensity Measurements

Fluorescence intensity measurements of the samples containing 3′-fluorescein-labelled oligonucleotide 5′-ACCCTGAAGTTCCGGCAAGCTG-FAM-3′ were carried out using CLARIOSTAR microplate reader (BMG Labtech, Ortenberg, Germany) in black 96-well half-area microplates (Costar, Thermo Fischer Scientific, Waltham, MA, USA). Steady-state fluorescence spectra of fluorescein were recorded upon excitation at 495 ± 4 nm in the range 500–600 nm. The fluorescence intensity values at the maximum (518 nm) were taken for the calculations.

### 2.6. Oligonucleotide Binding by Hydrogels

A piece of a hydrogel (1.0 mg) containing **TGn** or **PGn** prepared as described in Section 2.3 was rinsed with deionized water and then 40 μL of PBS was added. Fluorescein-labelled oligonucleotide 5′-ACCCTGAAGTTCCGGCAAGCTG-FAM-3′ was added in portions (350 pmol in 5 μL PBS each), followed by the gentle stirring for 10 min at 25 °C. After each addition, fluorescence intensity of the supernatant was measured, as described above. The amount of free oligonucleotide in solution was calculated from the fluorescence intensity values at 518 nm using a calibration curve (Figure S2, Supplementary Materials) obtained for the same quantities of the oligonucleotide as being added to a hydrogel (*n(total)*). The amount of bound oligonucleotide *n(bound)* was calculated as *n(total)-n(non-bound)* and plotted versus *n(total)*. Binding profiles were fitted using a model derived from the Langmuir isotherm [27], fitting was considered satisfactory if *R*^2^ > 0.95; saturation values were extracted from the fitting.

### 2.7. Oligonucleotide Release from Hydrogels

A piece of a hydrogel (1.0 mg) containing **TGn** or **PGn** prepared as described in Section 2.3 was rinsed with deionized water and then 40 μL of PBS was added. Fluorescein-labelled oligonucleotide 5′-ACCCTGAAGTTCCGGCAAGCTG-FAM-3′ was added (2100 pmol in 30 μL PBS) followed by the gentle stirring for 10 min at 25 °C. Then fluorescence intensity of the supernatant was measured and the amount of bound oligonucleotide was quantified using a calibration curve as described in Section 2.6. The corresponding fluorescence intensity value was taken as a reference for the calculation of the amount of released oligonucleotide.

Oligonucleotide-containing hydrogel was rinsed with deionized water (3 × 50 μL). 50 μL of 10 mM phosphate buffer adjusted to the pH 4.5; 5.0; 5.5; 6.0; 6.5; 7.0 was added and the sample was incubated at 25 °C upon gentle stirring. Aliquots of the supernatant were taken at given time points within 48 h of incubation; their pH was adjusted to 7.0. The fluorescence intensity of samples was measured and the amount of released oligonucleotide was calculated.

## 3. Results and Discussion

### 3.1. Dendrimer Synthesis

**Figure 2:**
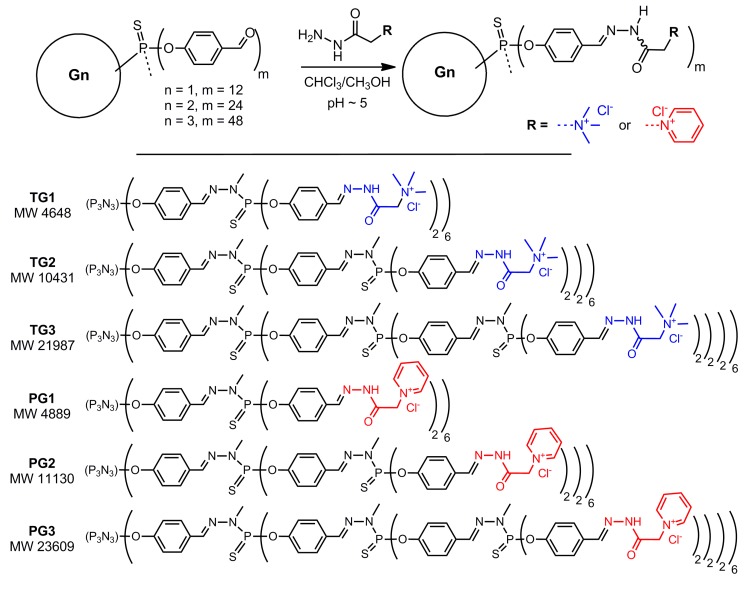
Synthesis and structures of acetohydrazone-terminated phosphorus dendrimers.

Cyclotriphosphazene core-based phosphorus dendrimers bearing trimethylammonium acetohydrazone (Girard T reagent) or pyridinium acetohydrazone (Girard P reagent) moieties on the periphery were synthesized by grafting of corresponding hydrazides onto the surface of aldehyde-terminated dendrimers upon mild acid catalysis [22]. The presence of an aromatic fragment on in the vicinity to the hydrazone stabilizes the Schiff base formed. The functionalized dendrimers were obtained in quantitative yield as mixtures of inseparable isomers around the hydrazone fragment –CH=N–NH–C(O)–CH_2_–, as revealed by ^1^H and ^13^C NMR spectroscopy. The structures of these dendrimers are given in Figure 2.

### 3.2. Gelation Studies

Girard-modified dendrimers possess numerous =N–NH–C(O)– fragments on the periphery and thanks to that, they are able to self-assemble into hydrogels. The association of dendrimers into hydrogel occurs by the hydrogen bonding between hydrazone groups on the surface of different dendrimers’ branches. To achieve that, dendrimer solutions were incubated at 65 °C that induces dendrimer hydration and intermolecular interactions. The hydrogels formed are thus classified as physical gels, suggesting that the gel network is potentially reversible.

The gelation is greatly facilitated by the use of hydrophilic additives that contribute to the hydrogen bonding network linking dendrimers together more efficiently. In particular, metal salts, Tris base, EDTA, ascorbic acid and so forth were reported to dramatically decrease the gelation time [22]. Herein, we used biocompatible additives, glucose, glycine and polyethylene glycol-4000, to assist the gelation. Gelation time and properties of the gels were found to depend on the dendrimer generation, structure of the acetohydrazone on the surface of dendrimer and on the nature of the additive. Table 1 summarizes the data on the properties of dendrimer hydrogels obtained. The formation of a rigid, coherent gel was considered to be complete gelation, otherwise incomplete (gel and turbid supernatant) or partial gelation (incoherent gel). In general, dendrimers **PGn** bearing pyridinium groups on the periphery appear to be better gelators than dendrimers **TGn**, that is in agreement with our previous findings [28]; glucose drives the faster formation of coherent, rigid gels. In the absence of additives, hydrogels were incoherent and in some cases, no gelation was observed within two weeks of incubation. It is worth noting that continuous incubation at relatively high temperature (65 °C) leads to the maturation of hydrogels resulting in their condensation and water loss [29]. Such gels are more rigid, the dendrimer content is higher than in others. Examples of dendrimer hydrogels are shown in Figure S1 (Supplementary Materials).

### 3.3. TEM Studies of Hydrogels

We tried to understand the three-dimensional structure of hydrogels by analysing two groups of transmission electron microscopy (TEM) data obtained by independent TEM methods (negative staining and ultrathin sectioning). We supposed that the TEM would allow to reveal the structure of hydrogels, to establish their structural units as well as to get the idea of the hydrogel alignment (porosity of gel network, alignment of gel fibres etc.). To the best of our knowledge, this is the first complete TEM characterization of dendrimer hydrogels: both structural elements and bulk morphology are shown.

**Figure 3:**
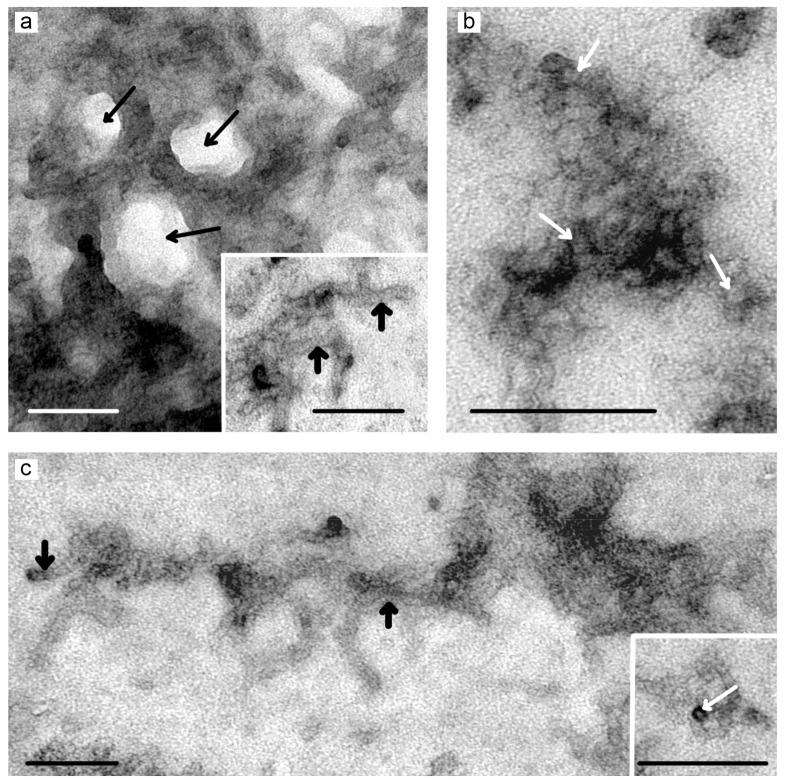
TEM images of hydrogels formed from dendrimer solutions in water: **TG2**@10% Glc (**a**,**b**), **PG2**@10% Glc (**c**). Spherical particles (white arrows) and fibres (thick black arrows) are shown. The three-dimensional dendrimer network with pores (thin black arrows) formed by spherical particles and fibres as well as aggregates of spherical particles (**b**) are observed. Negative staining with phosphotungstic acid. Scale bars represent 100 nm.

The study of gel samples adsorbed onto the TEM grid followed by standard negative staining with phosphotungstic acid (see Section 2.4) permitted to observe common structural elements of dendrimer hydrogels. Two types of structural units have been found: spherical particles and fibres (Figure 3). Both types consist of dendrimer molecules co-aggregated with additives. Spherical particles have high electron density after staining; their mean diameter is 8–10 nm. In the hydrogel network, they are associated in several layers being in close contact with each other. The fibres have low to moderate electron density, less associated; they act as cross-links between spherical particles. Spherical particles, their associates and fibres are co-assembled into a three-dimensional porous network.

To understand the alignment of dendrimers into hydrogel networks better, hydrogel samples were fixed by the paraformaldehyde cross-linking. Such a treatment permits to preserve the structure of complex objects; thanks to that, it became a standard technique for the preparation of biological samples for the TEM study. The following treatment does not distort the structure of the samples (see Section 2.4); ultrathin sectioning permits to observe even small objects, including supramolecular associates. The analysis of the consecutive sections provides the idea of the three-dimensional structure of samples.

**Figure 4:**
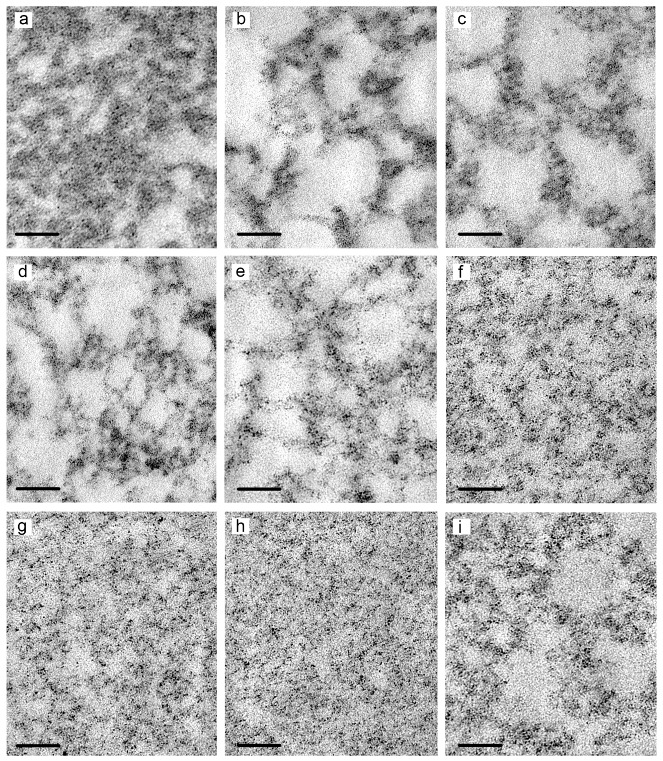
TEM images of hydrogel networks formed from dendrimer solutions in water: **TG2**@10% Glc (**a**,**b**); **TG3**@10% Glc (**c**); **PG2**@10% Glc (**d**); **PG3**@10% Glc (**e**); **PG1**@10% Gly (**f**); **PG2**@10% Gly (**g**); **PG3**@10% Gly (**h**); **PG3**@10% PEG (**i**). Ultrathin sections (~70 nm thick). Scale bars represent 100 nm.

Examination of hydrogel ultrathin sections (~70 nm thickness) has shown that all the gels under study have homological network structure differing by the pore size (Figure 4). The bigger were the electron-transparent regions in hydrogels (pores), the higher was the electron density of the network elements. The distribution of electron-transparent regions in samples correlates with the dendrimer content in hydrogels. Dendrimer generation contributes less in the density and morphology of gels.

Thus, the combination of two independent TEM methods (negative staining and ultrathin sections) permits to observe the structural elements of dendrimer hydrogels, visualize their alignment into a three-dimensional structure as well as qualitatively estimate differences in the morphology and structure of different hydrogels. The data obtained suggest that the gelation starts from the formation of dendrimer associates in solution further aggregating into developed networks composed of spherical particles and fibres. The TEM data are in good agreement with previously obtained SEM and cryo-TEM data [22]. Nevertheless, further physico-chemical and structural characterization of hydrogels would be needed to get more detailed information on the porosity of gel network, pore size, thickness and length of gel fibres, regularities of their alignment into a hydrogel and so on.

### 3.4. Oligonucleotide Binding and Release

Since Girard reagents have quaternized nitrogen atoms, dendrimer hydrogels have strong positive surface charge. This feature was used to load hydrogels with a model cargo. It would be interesting to test the strength of electrostatic forces for the drug loading, that is why an oligonucleotide, namely oligodeoxyribonucleotide 5′-ACCCTGAAGTTCCGGCAAGCTG-3′, has been used to study the hydrogel loading. 3′-Fluorescein-labeled oligonucleotide was added portionwise to hydrogels until the saturation is reached. At each step of the oligonucleotide addition, the amount of bound oligonucleotide was estimated from the residual fluorescence intensity.

**Figure 5:**
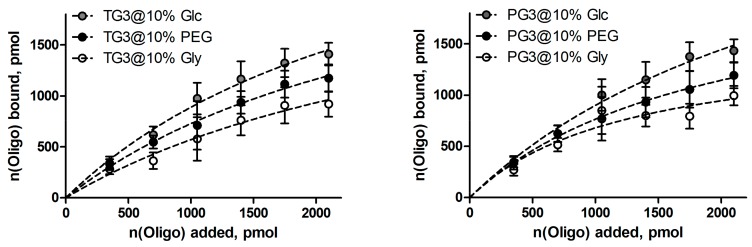
Profiles of the oligonucleotide binding by **TG3** and **PG3** dendrimer-based hydrogels.

In general, hydrogels bound oligonucleotides quite efficiently, with the efficiency correlating with the density of the hydrogel network. Figure 5 shows representative binding curves obtained for dendrimers **TG3** and **PG3**. Binding profiles were fitted using Langmuir binding models, the saturation values (i.e., the highest amount of oligonucleotide that can be bound) were estimated from the fitting curves. The cargo loading capacity was 1.5–3 μmol/g depending on the type of dendrimer and additive.

**Figure 6:**
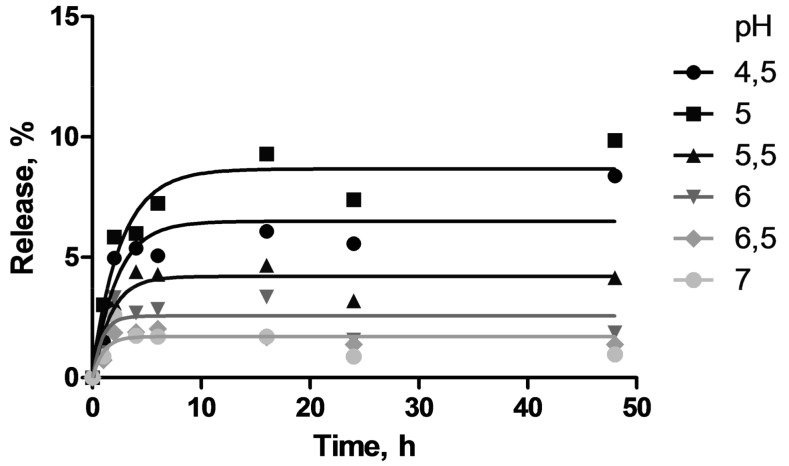
Kinetic profiles of the release of the oligonucleotide from the hydrogel **6G3**@10% Glc at a different pH.

An important aspect of the design of drug-containing biomaterials is the rate of cargo release. Indeed, the rate and completeness of the release define potential applications of materials. Here, oligonucleotide-saturated hydrogels were incubated in 10 mM phosphate buffer at pH 4.5–7.0 to examine the release of the cargo at the pH occurring in different tissues. Oligonucleotide-containing aliquots were taken at given time points within 48 h of incubation and the percentage of released oligonucleotide was calculated from the fluorescence measurements. The representative release kinetics plots are given in Figure 6.

The rate of release does not vary much with the pH change, the amount of free oligonucleotide in solution increases during the first 16 h of incubation and then stabilizes. Nevertheless, the oligonucleotide release appeared to be pH-dependent, the release efficiency increases at pH < 6. Unfortunately, even at acidic pH, the overall amount of the released oligonucleotide does not exceed 10%. This is likely explained by multiple electrostatic interactions between oligonucleotide molecules and hydrogel surface. It should be noted that in real hydrogel-tissue interfaces, the rate and efficiency of cargo release can be different due to the presence of other biopolymers displacing oligonucleotide from the gel.

## 4. Conclusions

In this work, physical hydrogels of phosphorus dendrimers containing biocompatible additives have been obtained for the first time. The hydrogels have well-developed network structure, with elementary units of these networks being distinctly observed by TEM. Hydrogels efficiently bound oligonucleotides, however, their release is slow. This may occur due to numerous cationic groups exposed to the surface of hydrogels. The use of gels of less charged dendrimers may allow to control the cargo release rate better.

It is interesting to note that the gelation time and hydrogel properties depend on the nature of an additive, however, oligonucleotide binding and release are defined presumably by the dendrimer percentage in a final gel. It would be interesting to study the biological effects of hydrogels containing different additives on different cell lines. Mechanistically speaking, due to the high surface charge, dendrimer hydrogels could increase the cell adhesion [30] thus serving as matrices for directed cell growth and development.

In summary, hydrogels based on cationic acetohydrazone-functionalized phosphorus dendrimers hold considerable interest and prospects for nanomedicine as potential biomaterials for tissue engineering and drug delivery. Our preliminary data suggest that physico-chemical and mechanical properties of the hydrogels as well as their biocompatibility and interactions with cell cultures and tissues deserve further investigation.

## Figures and Tables

**Table 1:** Properties of dendrimer hydrogels.

Composition (Dendrimer, Dispersant) ^1^	Gelation Time, h	Dendrimer Content, wt %	Hydration (10^3^ Water Molecules Per Dendrimer Molecule)	Appearance and Properties
**TG1**				
10% PEG in water	350	Incomplete gelation		White rigid gel
10% Glc in PBS	260	6.7	3.3	White rigid gel
10% Gly in PBS	260	4.0	5.6	White rigid gel
**TG2**				
10% Glc in water	60	2.3	22.3	White rigid gel
10% Gly in water	350	Partial gelation		Light yellow transparent gel
10% PEG in water	350	Incomplete gelation		White rigid gel
10% Glc in PBS	60	2.6	19.2	White rigid gel
10% Gly in PBS	350	Partial gelation		Light yellow gel
**TG3**				
10% Glc in water	60	2.4	44.4	White rigid gel
10% Gly in water	350	Partial gelation		Light yellow gel
10% PEG in water	330	5.9	17.6	Light yellow transparent rigid gel
PBS	350	Partial gelation		Light yellow gel
10% Glc in PBS	60	2.8	38.9	Milky white rigid gel
10% Gly in PBS	105	5.8	18.2	White rigid gel
10% PEG in PBS	350	Incomplete gelation		White rigid gel
**PG1**				
Water	350	Partial gelation		Light yellow transparent gel
10% Glc in water	350	Incomplete gelation		Yellow rigid gel
10% Gly in water	105	4.2	5.2	Light yellow transparent gel; semifluid, sticky
10% PEG in water	260	4.2	5.6	Light yellow transparent gel
PBS	350	Incomplete gelation		White rigid gel
10% Glc in PBS	60	2.6	9.0	Milky white rigid gel
10% Gly in PBS	60	2.7	8.8	Light yellow transparent gel; incoherent
10% PEG in PBS	230	6.7	3.4	Milky white rigid gel
**PG2**				
Water	350	Partial gelation		Colourless gel
10% Glc in water	60	2.5	21.6	Milky white rigid gel
10% Gly in water	135	4.8	11.0	Light yellow transparent rigid gel
10% PEG in water	230	3.1	17.6	Light yellow rigid gel
PBS	60	2.8	21.5	Milky white rigid gel
10% Glc in PBS	60	2.9	18.8	Milky white rigid gel
10% Gly in PBS	60	2.7	20.0	Light yellow transparent gel; incoherent
10% PEG in PBS	350	Incomplete gelation		White rigid gel
**PG3**				
Water	350	Partial gelation		Colourless gel
10% Glc in water	60	2.5	45.6	White rigid gel
10% Gly in water	90	3.4	33.3	Light yellow transparent rigid gel
10% PEG in water	350	Incomplete gelation		Colourless gel
PBS	60	2.8	40.4	Milky white rigid gel
10% Glc in PBS	70	2.7	42.7	Milky white rigid gel
10% Gly in PBS	60	2.8	40.4	Light yellow transparent rigid gel
10% PEG in PBS	280	2.2	51.9	Milky white rigid gel

^1^ Glc—glucose; Gly—glycine; PEG—polyethylene glycol-4000.

## Supplementary Materials

Supplementary Materials are available online at http://www.mdpi.com/1999-4923/10/3/120/s1.
