# Supplementary material for: Hydrogels of Polycationic Acetohydrazone-Modified Phosphorus Dendrimers for Biomedical Applications: Gelation Studies and Nucleic Acid Loading
Source: Pharmaceutics. 2018 Aug 6;10(3):120. doi: 10.3390/pharmaceutics10030120 (PMC6161142; doi:10.3390/pharmaceutics10030120)
Supplement: Supplementary file 1 [file pharmaceutics-10-00120-s001.pdf]

Supplementary Materials: Hydrogels of Polycationic Acetohydrazone-modified Phosphorus Dendrimers for Biomedical Applications: Gelation Studies and Nucleic Acid Loading

Evgeny K. Apartsin, Alina E. Grigoryeva, Audrey Malrin-Fournol, Elena I. Ryabchikova, Alya G. Venyaminova, Serge Mignani, Anne-Marie Caminade, Jean-Pierre Majoral

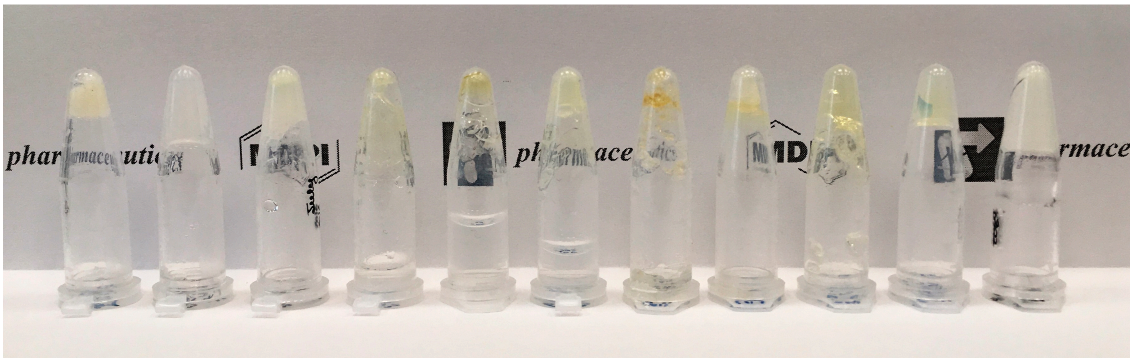

Figure S1. Examples of dendrimer hydrogels. From left to right: **TG1**@ 10% Glc in PBS; **TG3**@ 10% Glc in PBS; **PG3**@ 10% Gly in PBS; **PG3**@ water; **PG2**@ water; **PG3**@ 10% PEG in water; **TG2**@ 10% Gly in water; **TG2**@ 10% Gly in PBS; **PG2**@ 10% Gly in water; **TG3**@ 10% PEG in water; **PG3**@ 10% PEG in PBS.

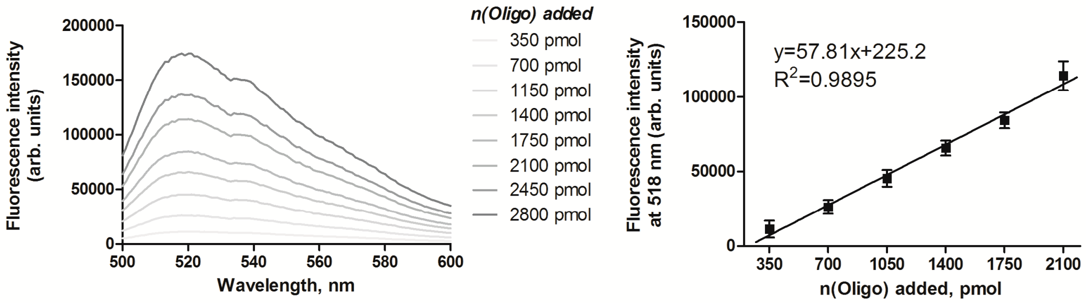

Figure S2. Fluorescence spectra of the oligonucleotide 5′-ACCCTGAAGTTCCGGCAAGCTG-FAM-3′ (left) and calibration curve for the calculation of the amount of bound oligonucleotide plotted based on these spectra (right).
